# Five-year follow-up of a posterior chamber phakic intraocular lens with a central hole for correction of myopia

**DOI:** 10.1007/s10792-023-02896-8

**Published:** 2023-11-07

**Authors:** Christoph Lwowski, Karel Van Keer, Tim Ruscher, Luisa Van Keer, Mehdi Shajari, Thomas Kohnen

**Affiliations:** 1 Department of Ophthalmology, Goethe-University, Theodor-Stern-Kai 7, 60590 Frankfurt Am Main, Germany; 2 Department of Ophthalmology, Ludwig Maximilian-University, Munich, Germany; 3 Department of Ophthalmology, UZ Leuven, Leuven, Belgium

**Keywords:** pIOL, ICL, ICL V4c, Myopia, Phakic

## Abstract

**Purpose:**

To evaluate intermediate and long-term visual outcomes and safety of a phakic intraocular posterior chamber lens with a central hole (ICL V4c) for myopic eyes.

**Methods:**

Retrospective, consecutive case study of patients that uneventfully received a ICL V4c for myopia correction, with a 5-year postoperative follow-up. Department of Ophthalmology, Goethe University Frankfurt, Germany.

**Results:**

From 241 eyes that underwent ICL implantation, we included 45 eyes with a mean age at surgery of 33 years ± 6 (18–48 years), with a 5 years follow-up. CDVA improved from 0.05logMAR ± 0.15 CDVA preoperatively to  − 0.00 ± 0,07 at 5 years and did not change significantly from 3 to 5 years’ time (*p* = 0.266). The mean spherical equivalent (SE) improved from -10.13D ± 3.39 to  − 0.45D ± 0.69. The change in endothelial cell count showed a mean decrease of 1.9% per year throughout the follow-up. Safety and efficacy index were 1.16 and 0.78, respectively. Cataract formation was seen in 2 of 241 eyes (0.8%), but in none of the 45 eyes that finished the 5-year follow-up.

**Conclusions:**

Our data show a good intermediate and long-term stability, efficiency, and safety of ICL V4c phakic lenses in myopic eyes comparable to other known literature.

**Supplementary Information:**

The online version contains supplementary material available at 10.1007/s10792-023-02896-8.

## Background

Myopia is a major issue in modern ophthalmology. A rising prevalence of this refractive error has been noted in Asia [1], as well as an increase in myopic patients in Europe [2]. Studies have shown that early onset of myopia can result in higher myopia in adulthood [3]. The rise in childhood myopia we are currently witnessing is thus expected to further add to the problem of high myopia in adults.

While corneal laser surgery as laser in situ keratomileusis currently remains the most common procedure for surgical correction of myopia [4], it has its natural limitations by residual stromal thickness to provide sufficient corneal stability after the treatment [5, 6]. Refractive lens exchange offers such alternative, but the resulting loss of accommodation makes this treatment a less ideal option for younger patients, and there is an increased risk of retinal detachment, especially in myopic patients [7], which is not reported in patients receiving phakic IOLs (pIOL) [8].

The implantation of a phakic intraocular lens (pIOL) could offer a better option since it is able to correct high refractive errors without losing optical quality or accommodation and poses only a moderate risk profile due to its minimally invasive implantation technique [9, 10]. The implantable collamer lens (EVO Visian ICL, Staar Surgical, CA, USA), in particular, were able to position itself as one of the most implanted pIOLs. It is implanted behind the iris in the sulcus in front of the crystalline lens. This position reduces the risk of endothelial cell loss compared to anterior chamber pIOLs [11] but has caused concerns regarding inducing cataract and elevated intraocular pressure [12]. To improve the flow of intraocular fluid, a Nd:YAG iridotomy was needed. The ICL V4c with a central hole (CentralFLOW Technology) was developed to overcome this limitation [13]. The short and intermediate safety and efficacy with the ICL V4c has already been demonstrated in various trials [14, 15] but because it has only been on the market little above 10 years, long-term results are still rare. These lenses are typically implanted in young adults, meaning long-term data on endothelial cell behavior, cataract formation, and risk for glaucoma are of main interest for patients and surgeons.

This trial was conducted in the Department of Ophthalmology of a large university clinic in Germany that specializes in refractive surgery with the purpose of evaluating the intermediate and long-term results in otherwise healthy patients that received implantation of an ICL V4c.

## Methods

The study protocol was approved by the local Ethics Committee of the University Frankfurt, Germany and followed the tenets of the Declaration of Helsinki.

The medical records of all patients that received an ICL V4c from January 2013 to December 2018 were screened for patients with myopia.

Exclusion criteria were pathologies that could possibly limit postoperative visual acuity or the calculation of the pIOL (e.g., corneal scaring or keratoconus). Patients were included if they did not match any of the criteria above and if postoperative data were available for 3 years and 5 years postoperatively. Only eyes with successful mono- or bimanual aspiration of the viscoelastic fluid were included. Five of the first eyes had an anterior subcapsular cataract due to viscoelastic removal via forceful irrigation and injection of BSS and were therefore excluded [16] Patients of 18 years or older were included, since this is the age recommended by the German Commission of Refractive Surgery (KRC) for pIOL implantation in their 2022 guide lines [17].

### Preoperative and postoperative assessments

Axial length (AL), anterior chamber depth (ACD), lens thickness (LT), and white-to-white distance (WTW) were collected with a partial coherence interferometer integrated in the Pentacam AXL (Oculus, Wetzlar, Germany) and the IOL Master 500 / 700 (Zeiss Meditec, Jena, Germany). The simulated keratometry (sim K) was measured with the IOL Master 500 or 700. Current literature shows that the measurements of the IOL master 500 and 700 are comparable [18]. Preoperative and postoperative subjective refraction and visual acuity were performed by an optician (SE; in diopters = D, 6 m lane, EDTRS charts). The postoperative vault, and pre and postoperative corneal astigmatism, was measured with Scheimpflug tomography (Pentacam, Oculus, Germany). Endothelial cell count (ECC) was automatically evaluated with the CEM530 (NIDEK Inc., Japan). The intraocular pressure was measured by an ophthalmologist using Goldmann applanation tonometry. Scheimpflug and ECC measures were performed in the same room at 0.1–0.2 lx (low mesopic).

### Lens and surgery

The ICL V4c is an implantable collamer lens made from a proprietary hydroxyethyl methacrylate/porcine collagen-based biocompatible polymer material and ultraviolet absorbing chromophore. It features a convex-concave optic of 4.9–5.8 mm and a 380 µm hole to improve the flow of aqueous humor. The overall diameter of the ICL V4c varies according to the anatomy of the patient’s eye (primarily WTW and ACD) and the manufactures formula and comes in four sizes (12.1, 12.6, 13.2, and 13.7 mm).

The ICL V4c is available from  − 0.5D to  − 18.0D and for astigmatism of up to 6.0D. Patients need to have a ACD (from endothelium) of at least 2.80 mm. All surgeries were performed by a single, experienced surgeon (TK). A clear corneal incision was made temporally with a 2.6 mm steel knife and one paracentesis. This incision was used to install the ophthalmic visco-surgical device or manipulate the ICL. The ICL was implanted in the sulcus. Incisions were hydrated and a local therapy with steroid (dexamethasone 1.3 mg/ml, four times per day, for 2 weeks) and NSAID (nepafenac 3 mg/ml, once a day, for 6 weeks) eye drops was conducted.

ICL calculations were performed together with Staar Surgical using a modified vertex formula that includes preoperative manifest refraction, cycloplegic refraction, keratometric data, corneal thickness, and ACD.

### Statistics

The sample size estimation for this retrospective trial was performed with the G*Power 3.1 Software (Heinnrich Heine University Cologne, Germany). To prove an effect size of 0.5, which would be a difference in visual acuity of 0.05 logMAR with a standard deviation of 0.1 logMAR, with *α* = 0.05 and power 1 − *β* = 0.9, a sample size of 35 eyes was needed.

A Kolmogorov–Smirnov test was used to access if data was normally distributed. For multiple testing of continuous variables, a repeated-measures ANOVA or Friedman test was used and if a significant difference was found, a post hoc analysis with paired t-test or Wilcoxon signed-rank test was performed to determine between which time points the significant difference occurs. If needed P values were Bonferroni corrected, otherwise a *P* value of < 0.05 was considered statistically significant. Statistical analysis was performed using Excel 2011 (Version 14.7.7; Microsoft) and SPSS (Version 26.0, IBM).

Data are reported as recommend by the Journal of Cataract and Refractive Surgery [19] and Journal of Refractive Surgery [20] including the standard figures for reporting visual results of refractive surgery.

## Results

### Preoperative data

From 2013 to 2018, a total of 241 eyes received an ICL V4c for myopia at a mean age of 33 years ± 6 (18–48 years). Mean follow-up time of these eyes was 34 days ± 50. For 45 eyes, data of 3 and 5 years postoperative was available. The mean preoperative SE was  − 10.13D ± 3.39; the corrected visual acuity (CDVA) was 0.05logMAR ± 0.15. Since the preoperative uncorrected visual acuity (UDVA) was too low in some patients to express in logMAR, these measurements were not included. Mean preoperative endothelial cell count (ECC) was 2811/mm^2^ ± 375. Toric ICLs were implanted in 21 of the 45 eyes (46%). There was no significant difference in the follow-up between eyes with and without toric ICLs.

### Visual acuity and spherical equivalent

The visual acuity improved from 0.05logMAR ± 0.15 CDVA preoperatively to -0.02logMAR ± 0.09 at 3 and  − 0.00 ± 0,07 at 5 years, which was not statistically significant (p = 0.13) and did not change significantly from 3 to 5 years’ time (*p* = 0.221, supplemental Fig. 1). The corrected and uncorrected visual acuity can be found in Table 1. There was a decrease in UDVA over time (3–5 years, *p* = 0.017), but using a Bonferroni corrected *p* value (0.12) this was not statistically significant.

**Table 1:** pre- and postoperative data (*n* = 45)

	Preop	3 years	5 years	*p* value
UDVA (logMAR)	n.a	0.09 ± 0.20 (− 0.1–0.9)	0.15 ± 0.22 (− 0.1–0.8)	0.12
CDVA (logMAR)	0.05 ± 0.15 (− 0.2–0.8)	− 0.02 ± 0.09 (− 0.2–2)	0.0 ± 0.07 (− 0.1–0.2)	0.13
SE (diopter)	− 10.13 ± 3.39 (− 20.25–2.75)	− 0.15 ± 0.53 (− 2.5–1)	− 0.45 ± 0.69 (− 2.5–0.875)	< 0.001
IOP (mm Hg)	14.5 ± 3 (10–18)	14.7 ± 2.4 (10–19)	14.5 ± 2.9 (10–20)	0.501
ECC (x/mm^2^)	2811 ± 374 (1834–3878)	2649 ± 300 (1726–3236)	2520 ± 279 (1725–3294)	< 0.001

Data are represented as mean ± standard deviation (range)

UDVA, uncorrected distant visual acuity; CDVA, corrected distant visual acuity; SE, spherical equivalent; IOP, intraocular pressure; ECC, endothelial cell count

*p* values are Bonferroni corrected for multiple testing

**Fig. 1:**
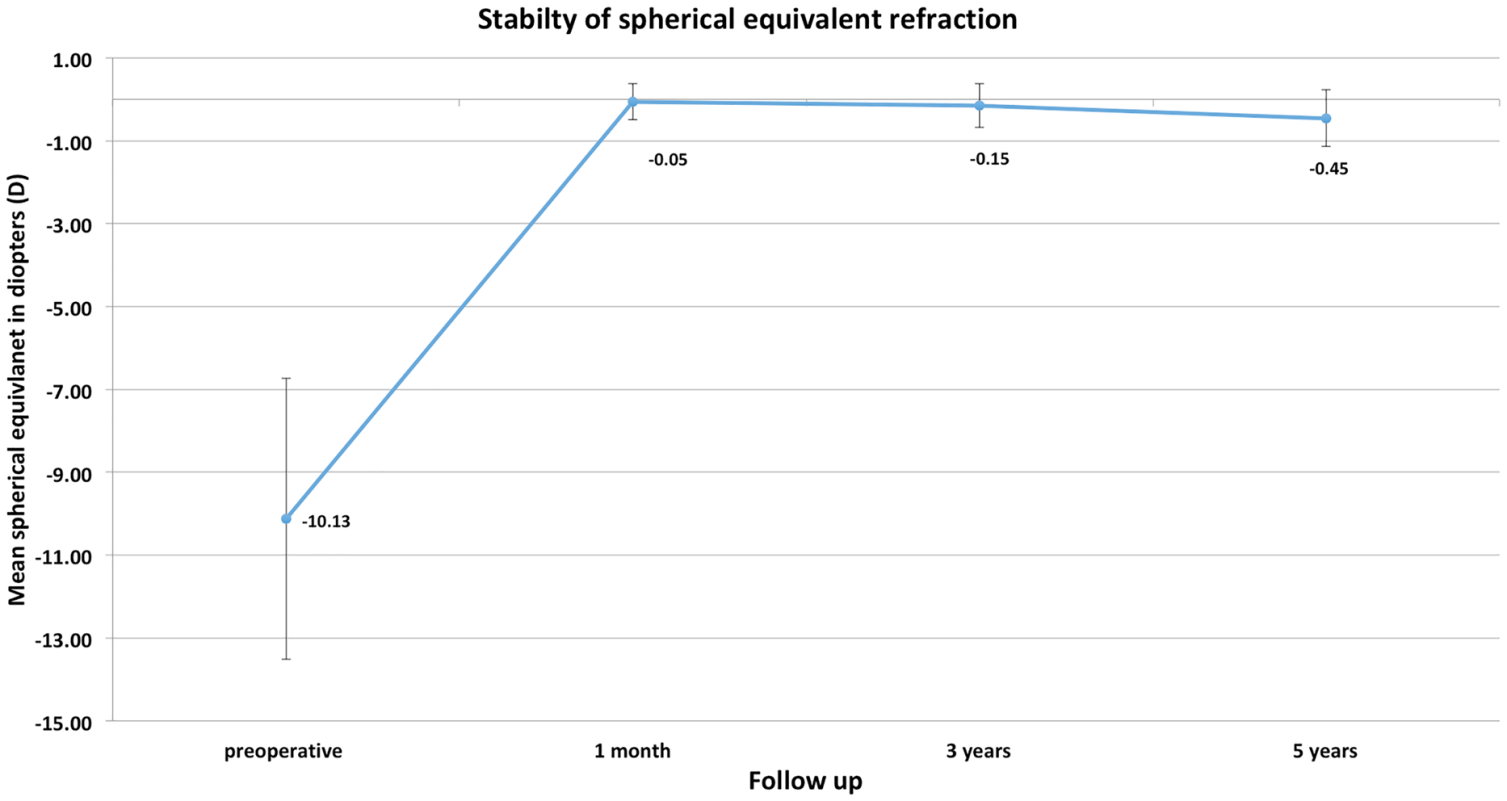
Stability of the spherical equivalent (SE) during the 5-year follow-up

**Fig. 2:**
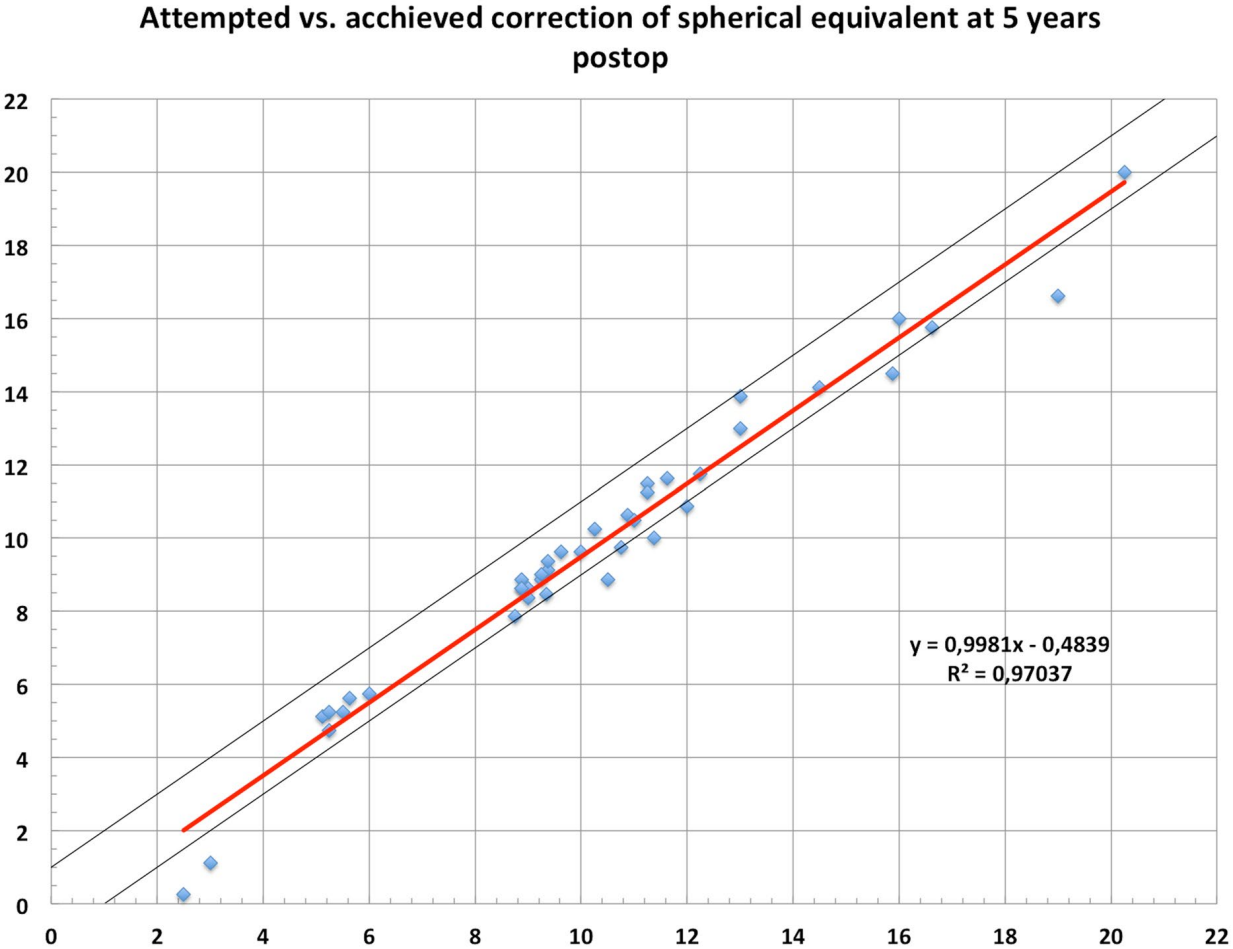
Attempted and achieved correction of spherical equivalent at 1 year postoperatively

**Fig. 3:**
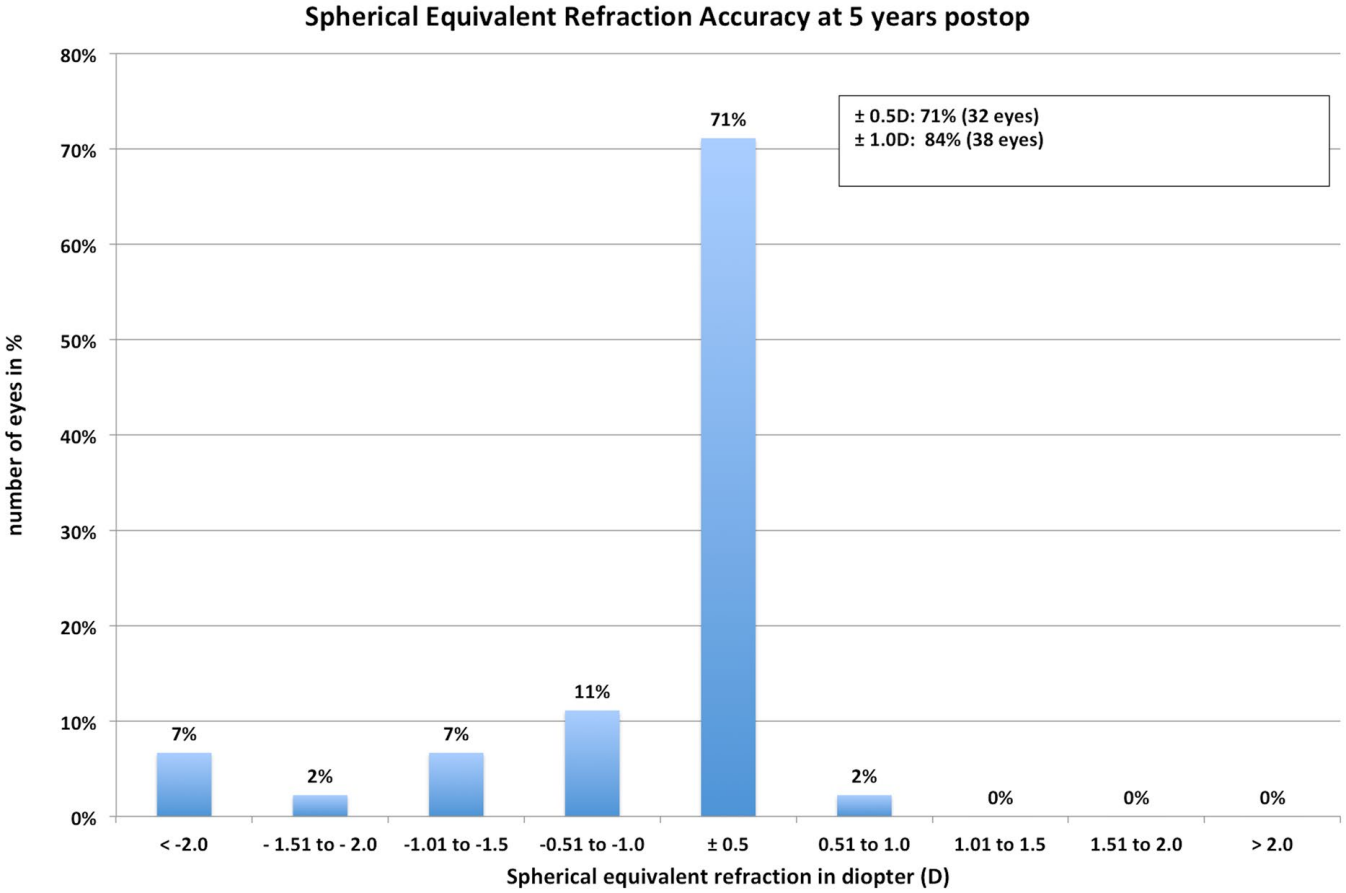
Cumulative residual spherical equivalent refraction (SE) at 1 year postoperatively

**Fig. 4:**
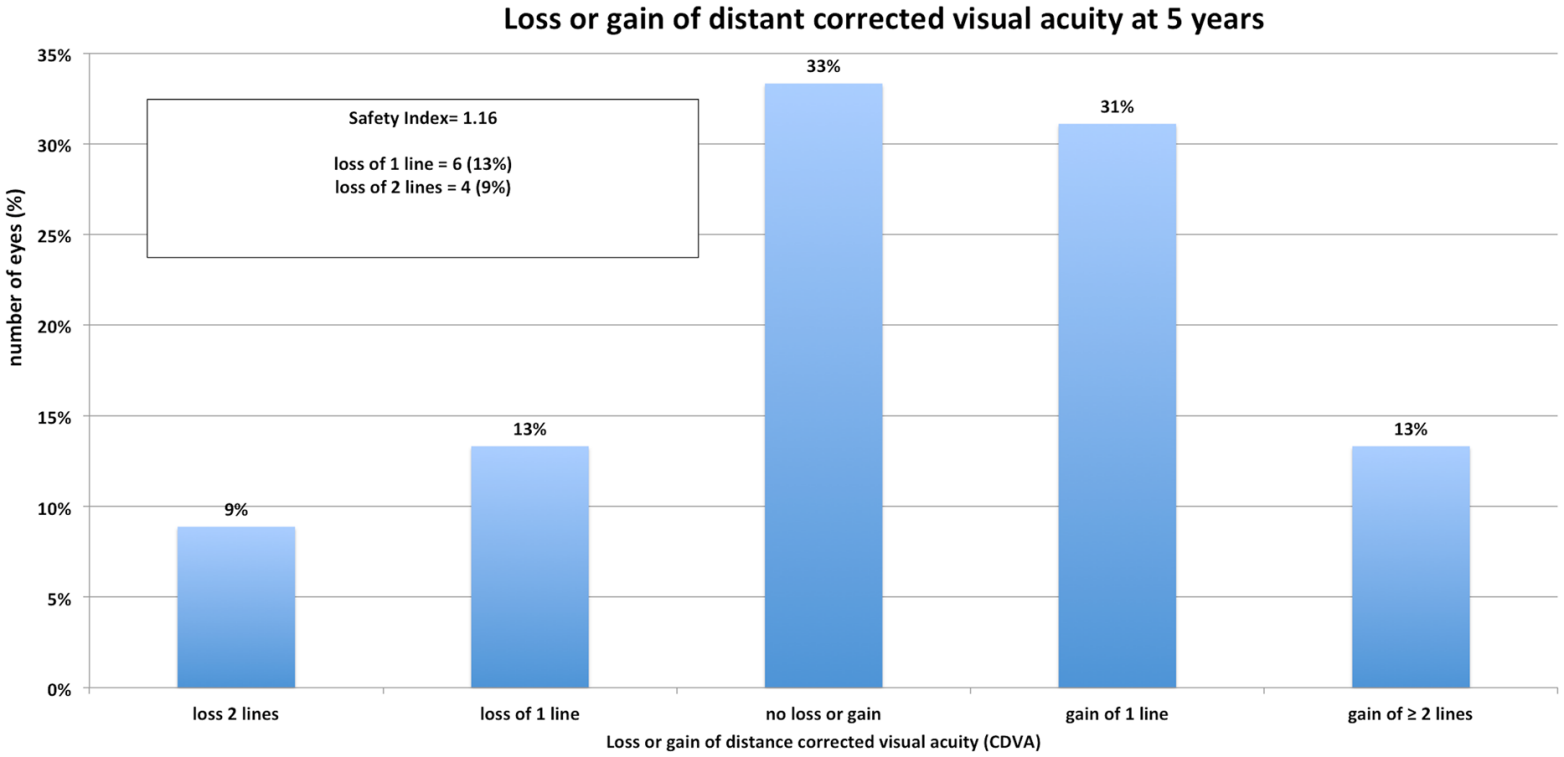
Loss or gain of corrected distant visual acuity (CDVA) from preoperative to 1 year postoperatively

The same does account for the postoperative mean spherical equivalent that improved to  − 0.05D ± 0.44 at 1-month postoperative and did have a slight trend toward myopization during the 5 years of follow-up ( − 0.15D ± 0.52 at 3 and  − 0.45D ± 0.69, Table 1, Fig. 1) but did not change significantly over time when using a Bonferroni corrected *p* value (0.266). However, comparing pre to postop SE shows a highly significant difference as expected (*p* < 0.001). The accuracy was very high even after 5 years with a *r*^2^ of 0.97 terms (Fig. 2). Seventy-one percent were within ± 0.5D and 84% within ± 1.0D after 5 years (Fig. 3). From the point off safety, 4 eyes (9%) lost 2 lines of CDVA while 44% gained at least one line of CDVA (Fig. 4) again at 5 years postop.

**Fig. 5:**
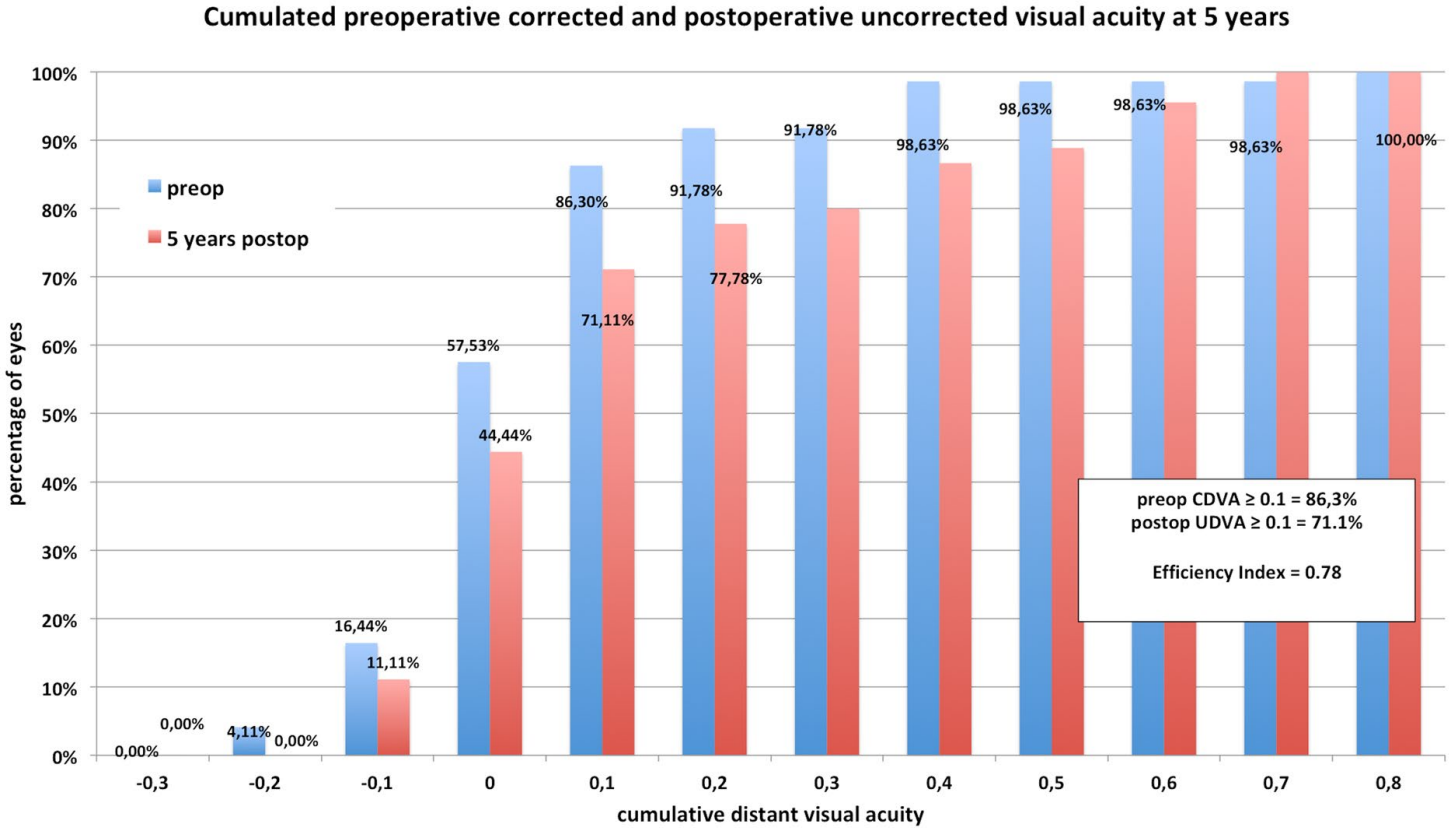
Cumulated corrected distance visual acuity (CDVA) from preop compared to uncorrected visual acuity (UDVA) 1 year postop

The safety and efficacy index at 5 years postoperatively was 1.16 (preop CDVA/postop CDVA) and 0.78 (preop CDVA/postop UDVA), respectively (Fig. 5). The EI after 1 month was 1.38 but decreased due to the mentioned myopization.

No patient underwent corneal laser enhancement surgery to compensate for postoperative refractive error.

### Endothelial cell count and intraocular tension

The endothelial cell count (ECC) did change over the time. The initial ECC was 2811/mm^2^ ± 375, 2649/mm^2^ ± 300 at 3 and 2520/mm^2^ ± 279 at 5 years (*p* < 0.001) postoperatively, which is an overall decrease of 9.5% or 1.9% per annum (supplemental Fig. 2). The post hoc analysis shows that the decrease was significant between pre- and 3 years postop (*p* = 0.005) but not between 3 and 5 years postop (*p* = 0.090). Four eyes (8.6%) had a ECC loss of above 25%, no eye fell below 1500/mm^2^ during the follow-up. A significant correlation between ACD and ECC loss was not found (Pearson coefficient = − 0.144).

The intraocular pressure (IOP) did not change from pre- to postoperative (*p* = 0.501) and is shown in Table 1 and Supplemental Fig. 3. In the early postoperative period, there were isolated cases of elevated IOP, up to 40 mmHg, presumably due to retained viscoelastic in the anterior chamber. With adequate topical treatment, none of these cases persisted for longer than 1 week postoperatively.

### Vault, ICL size, and cataract formation

The mean postoperative vault as measured by Scheimpflug imaging was 425 µm ± 204 with a range from 100 to 940 µm. Two eyes had a vault < 250 µm (hypo vault) and three > 750 µm (hyper vault). The mean ICL V4c diameter was 13.02 mm ± 0.34.

Cataract formation was not seen in the follow-up group. However, 2 eyes of one patient at 21 months from initial surgery and at an age of 50 years and with already preexisting lens inhomogeneity prior to ICL implantation did show cataract formation. Both were successfully treated by removal of the ICL and natural lens and IOL implantation. The vault for this patient is not known.

No other lens-related complications occurred in our patient collective during the follow-up.

## Discussion

We report the retrospective data from 45 eyes that received an ICL V4c implantation for correction of myopia in a major German university clinic over the time span of 7 years. We reviewed all patient charts and included those with a follow-up of 5 years including subjective refraction, uncorrected and corrected visual acuity, endothelial cell count, intraocular pressure, and assessment of possible complications. With a mean preoperative SE of  − 10.13D ± 3.39, we treated highly myopic patients and were able to correct them to emmetropia with a SE of 0.05D ± 0.44 at 1-month postoperatively with a slight trend of myopization in the long-term follow-up. This could be due to changes/growth of the natural lens with consecutive myopic shift of the refraction. Alternatively, a possible mechanism would be axial growth. But since no measurements were made, we cannot answer this.

Comparing our postoperative spherical equivalent to the current literature, Wan et al. [21] reported similar outcomes to ours in 2020 for 137 eyes 6 months postoperatively with a mean SE of  − 0.03D ± 0.07. Kamiya [22] reported a SE of 0.01D in eyes with myopia ≤ − 6D and 0.02D in highly myopic eyes > − 6.0D 12 months after the implantation of the ICL in more than 350 eyes. Additionally, the predictability outcomes were excellent with 93% and 94% of eyes being within ± 0.5D of target refraction. Kojima compared the V4c to the slightly bigger V5 ICL and had a postoperative SE of 0.05D ± 0.07 after 6 months for 23 patients [23]. These results do exceed ours, with 71% of eyes being within ± 0.5D and 94% being within ± 1.0D, but our results were taken 5 years postoperatively. Shimizu et al. [24] and Alfonso et al. [25] reported similar results with 95–98% of eyes being within 0.5D of target refraction. Five years results similar to ours are reported by Chen et al. [26] with 79% of eyes being within 0.5D of target. Some authors report more myopic outcomes in the long-term follow-up [27, 28]. Those more myopic outcomes could be due to a progressive myopization in young patients or changes of the lens nucleus that could be an early stage of cataract formation. This was the interpretation of the myopic outcome of  − 0.9D ± 0.95 by Yan et al. [29] after a 2 years follow-up. Other trials describe myopization as early as 1 year after ICL implantation [30]. With a myopic shift of  − 0.5D in our patients during our follow-up of 5 years, this is a trend that we were able to reproduce. When comparing long-term results, repeatability needs to be kept in mind in terms of efficacy and stability which can be seen in publications from Liu et al. [14, 31] and Alfonso et al. with a follow-up of up to 25 months and up to 5 years with only 69% and 67% of eyes being within ± 0.5D. Data of a 10-year follow-up by Choi et al. [32] report a myopic outcome with a mean SE of  − 0.69D and a UDVA that decreased from 0.06 logMAR to 0.13 logMAR accordingly at the end of the follow-up while CDVA remained stable.

Postop CDVA was  − 0.02logMAR ± 0.09 and stayed at this level during the 5-year follow-up. The uncorrected visual acuity was a little bit reduced due to the discussed myopization at 5 years postoperative. Comparing those results to Shimizu et al. in their trial at 6 months [24] and after 5 years [10], they report a better VA compared to our patients with a UDVA of  − 0.2 logMAR and  − 0.17 logMAR, respectively, and CDVA of  − 0.25 and  − 0.24 logMAR. But the patients in this trial did have a better CDVA preoperatively compared to our patients with  − 0.17 logMAR compared to 0.05 logMAR. Similar results are reported by Kojima et al. in 2018 [23]. Results comparable to ours are reported by Wan [21], Fernandez-Vega-Cueto [27], and Lisa [33].

Since ICLs are implanted in young patients, the long-term follow-up is of utmost importance due to axial length growth in highly myopic patients and possible changes in the refractive power of the lens. This is shown by the 5 years data of Alfonso et al. that showed a worse UDVA compared to ours (0.13 logMAR) but a similar CDVA (0.02logMAR) in 147 eyes [31]. Similar results are reported by Cao et al. [34] This was reproduced by our data as well with a UDVA of 0.15 logMAR after 5 years. But still some papers report a lower overall VA compared to our like Rizk et al. [35]

With 4 eyes (9%) losing 2 lines of CDVA but 44% gaining at least one line, we reached a very good safety index (SI 1.16) and sufficient efficacy index (EI 0.78) at 5 years postoperatively. This matches similar retrospective trials like Kamiya et al. [36], who report an EI of 1.18 and SI of 0.89 at 8 years postoperatively, with a loss of 1 line CDVA in 8% of eyes or Chen et al. [26] with SI of 1.03–1.32 and EI of 0.83–0.83 for different stages of myopia. These results are comparable to other trials of Chen et al. [28] or Martinez-Plaza et al. [37] Better SI and EI are reported by others with a SI of up to 1.67 [29] and EI of up to 1.5 [38] As already mentioned, the UDVA is influenced by the postoperative refraction that can change during the follow-up which could explain the EI of Fernandez-Vega-Cueto [27] or Alfonso [31] at 3 and 5 years postoperatively with 0.9 and 0.87 EI. However, when discussing the visual acuity of the follow-up, the retrospective nature of this trial must be considered. While patients in prospective trials will often be pushed to their visual limits and VA testing usually relies on forced choice testing, this could be a limitation of our trial and a possibly explanation of the number of eyes losing CDVA in our patients. But comparable results with CDVA loss of 1 or more lines in 17% of eyes were reported in other trials as well [38]. Packer et al. report even higher rates during their 11 years follow-up with 36% losing 1 line CDVA after 5 and 50% after 11 years, which could be due to lens opacification or corneal changes [39].

Due to the anterior chamber depth and possible changes in flow of the aqueous humor, the ECC needs to be monitored. In our study, the overall endothelial cell loss from preoperative to 5 years was 291 (9.66%), which would be 1.9% per year and therefore little above the range of a physiological cell loss in healthy eyes [40]. However, this could possibly be due to an initial cell loss caused by the procedure itself. Since we do not report short-term data, this cannot be verified by us. But initial cell loss due to the surgical trauma was seen in other trials, with a loss of 7.1% [41] after 1 year or a loss of 8.5% [25] after 6 months. After this initial loss of cells, most patients return to the physiological cell loss as described in the 5-year follow-up of Shimizu et al. [10] with a loss of 5.4% compared to an initial cell loss of 2.8% [24] for the first 6 months. Cell loss comparable to this was also published by Kohnen et al. in a 10-year follow-up after anterior chamber pIOL implantation [42]. This is an interesting finding since other papers report lower decreases of the ECC in ICL eyes with a cell loss of below 1% [15, 22]. But other extremes exist as well with Ganesh et al. [43] reporting a ECC loss of 9% after 1 year in 30 eyes or 22% after 5 years. With the mentioned cell loss of 1.9%/year, our results do compare to the literature as described and show that the ECC loss is above the range of the physiological loss of cells. Therefore, monitoring of the cell count is still highly important.

In our patients, a mean pIOL size of 13.02 ± 0.34 was implanted (mean WTW: 12.0 mm ± 0.40, mean ACD: 3.21 mm ± 0.31). The mean vault in our patients was 425 µm ± 204 with a range from 100 to 940 µm. This is similar to the current literature that reports vaults of 389 µm (90–700 µm) [14] or 405 µm (100–980 µm) [33]. However, the postoperative vault is also depending on pupil size, and this could possibly influence our data [44]. Still, all measures were taken in the same room at the same, low mesopic light conditions. New formulas developed to improve ICL calculation seem to reach better results, especially when using data of swept source OCT of the anterior segment [45]. Calculating the vault depends on different ocular parameters like, e.g., corneal diameter, anterior chamber depth, or axial length. Varying formulas are known and show varying results. Formulas depending on OCT seem to be the most promising [46]. With only 2 eyes having a hypo and 3 eyes having a hyper vault, the rate of eyes not being within the wished range is low in our trial. However, due to the retrospective nature of our trial, the timepoint of the vault being measured is rather inconsistent, which makes it hard to compare it to the current literature. If the vault is too small, the residual refraction could be myopic and vice versa. Additionally, it could cause cataract formation located at the anterior capsule of the lens. The rate of postoperative complications was very low in our patients. Two of the 241 eyes (0.8%) that had the pIOL implanted had cataract formation at a mean follow-up time of 21 months postoperatively which compares to or outperforms most studies that describe a rate of cataract formation below 5%, [35, 46] like a recent publication by Gonzalez-Lopez et al. [47] that found anterior cataract in one of 24 eyes (4.17%) with low vault. However, none of the eyes that finished the 5-year follow-up had a clinically significant cataract. Elevated tension was only seen at one week postoperative but could be treated by eye drops and did not increase compared to preoperative tension during the follow-up. This also is comparative to most trials [13, 15, 28, 31]. Chronic iritis and/or pigment dispersion was not seen in our patients but is described in other studies [35].

The main limitation of our trial is the retrospective nature of the study leading to an unstandardized postoperative follow-up time. However, thanks to strict postoperative standards, we believe the procedures and measurements that we reviewed are still comparable to current practices. Additionally, we only included procedures without complications.

## Conclusion

In our retrospective trial, we report on the results of myopic patients after implantation of an ICL V4c. Reviewing a mean follow-up time of 5 years, we were able to show that the pIOL provides a safe and effective tool for correcting myopia with excellent mid- and long-term results. Accurate estimation of the postoperative vault to select the optimal sizing is crucial to minimize the risk for long-term complications. Long-term follow-up for possible ECC loss or cataract formation remains important.

### Supplementary Information

Below is the link to the electronic supplementary material.

Supplementary file1 (TIFF 117 KB)

Supplementary file2 (TIFF 426 KB)

Supplementary file3 (TIFF 100 KB)

Supplementary file4 (DOCX 15 KB)
