# Supplementary figures and images for: Five-year follow-up of a posterior chamber phakic intraocular lens with a central hole for correction of myopia
Source: Int Ophthalmol. 2023 Nov 7;43(12):4933–43. doi: 10.1007/s10792-023-02896-8 (PMC10724086; doi:10.1007/s10792-023-02896-8)

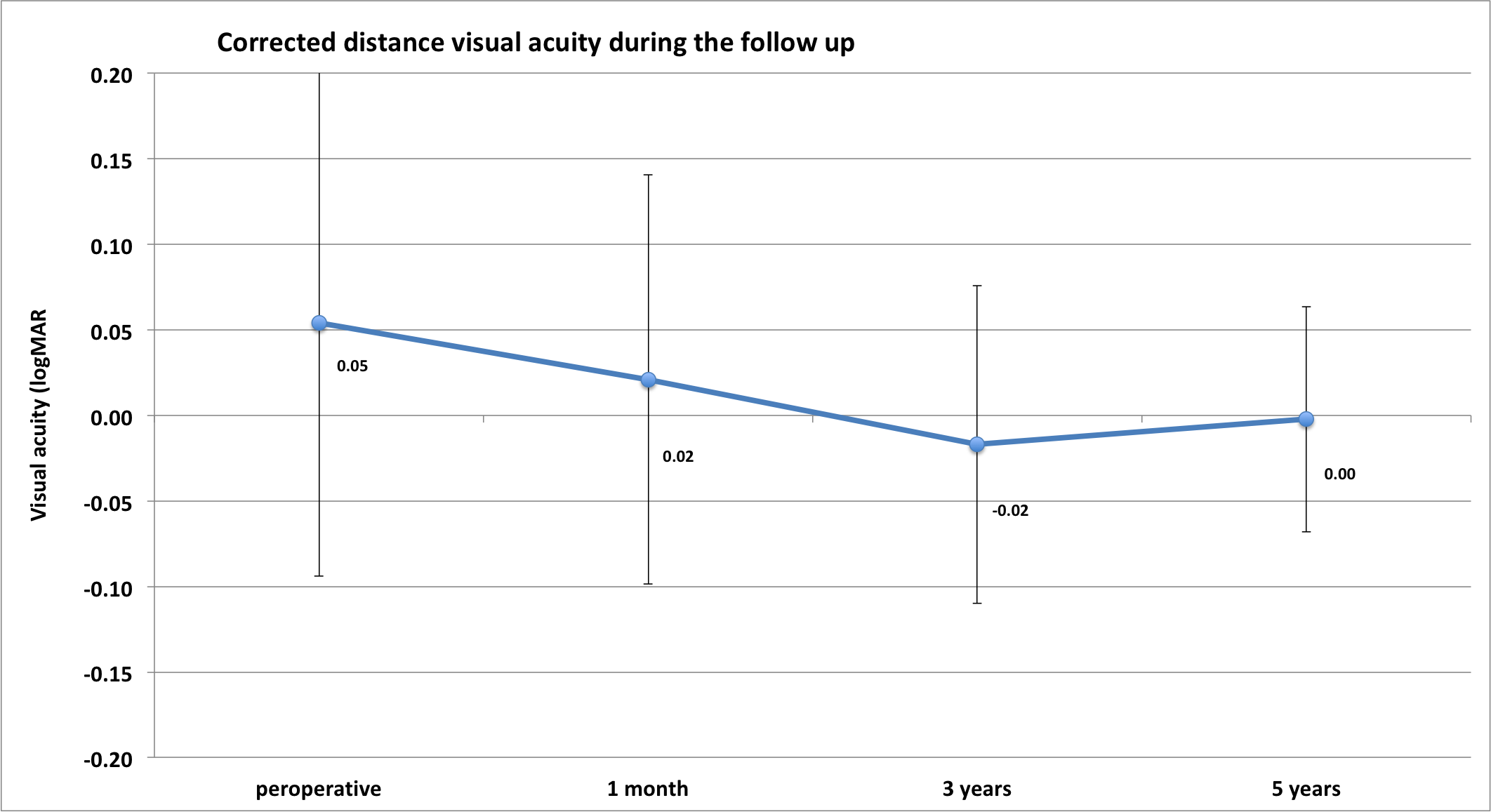

Supplement: Supplementary file 1 — Supplementary file1 (TIFF 117 KB) [file 10792_2023_2896_MOESM1_ESM.tiff]

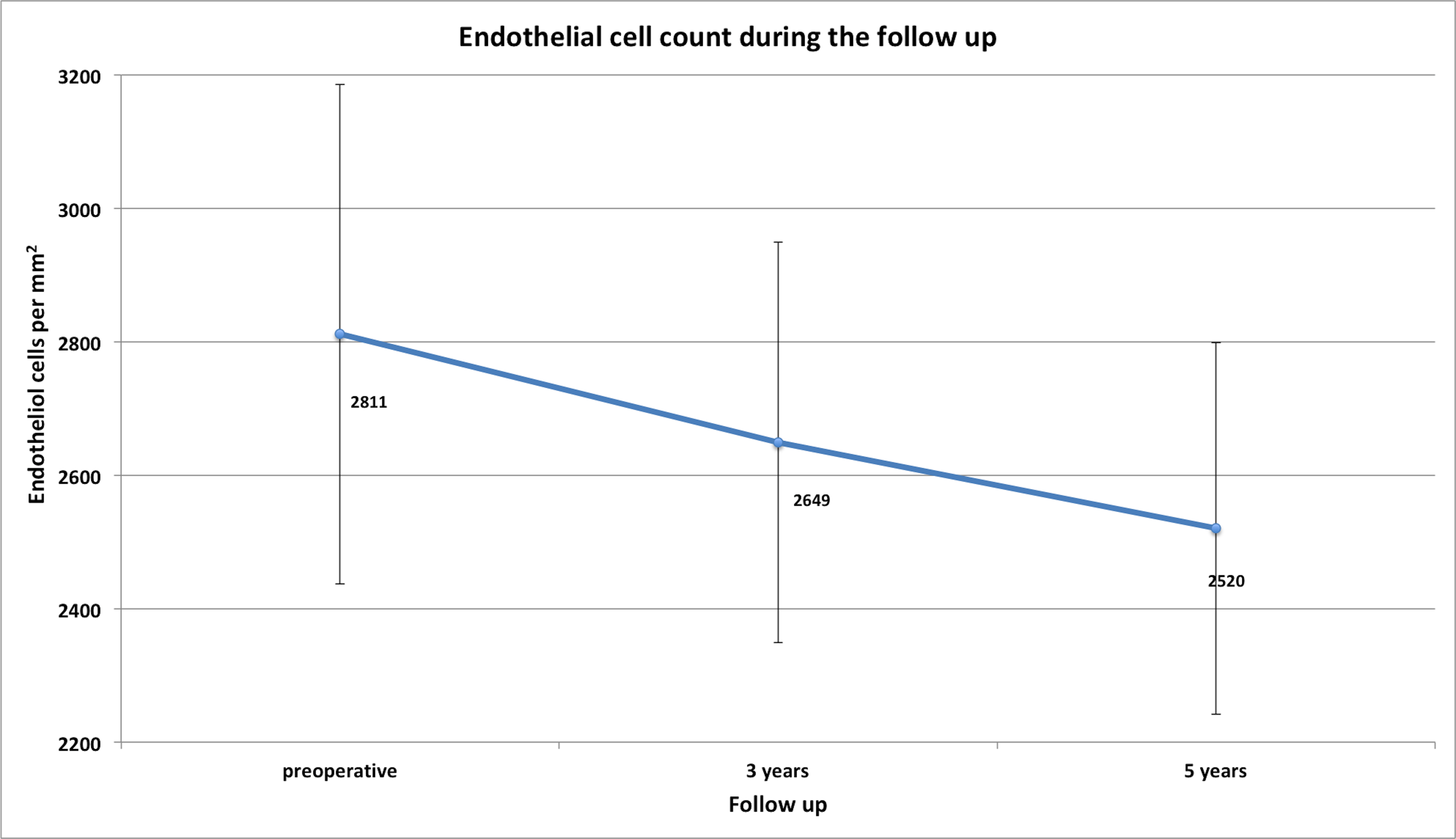

Supplement: Supplementary file 2 — Supplementary file2 (TIFF 426 KB) [file 10792_2023_2896_MOESM2_ESM.tiff]

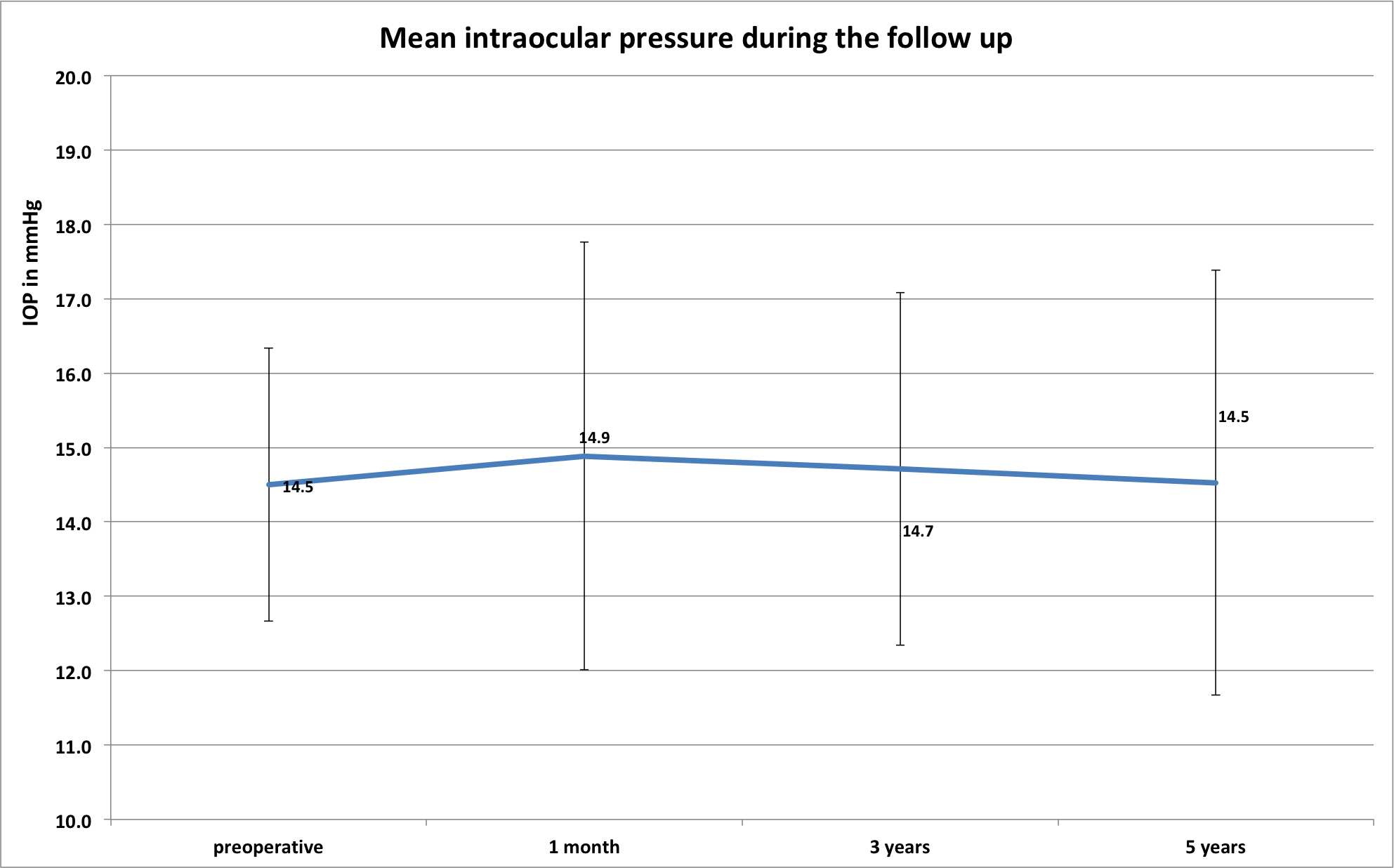

Supplement: Supplementary file 3 — Supplementary file3 (TIFF 100 KB) [file 10792_2023_2896_MOESM3_ESM.tiff]
