# Supplementary material for: Five-year follow-up of a posterior chamber phakic intraocular lens with a central hole for correction of myopia
Source: Int Ophthalmol. 2023 Nov 7;43(12):4933–43. doi: 10.1007/s10792-023-02896-8 (PMC10724086; doi:10.1007/s10792-023-02896-8)
Supplement: Supplementary file 4 — Supplementary file4 (DOCX 15 KB) [file 10792_2023_2896_MOESM4_ESM.docx]

Supplemental Figure 1: Corrected distant visual acuity (CDVA) during the 5 year follow up

Supplemental Figure 2: Endothelial cell count per square millimeter (ECC x/mm2) during the 5 year follow up

Supplemental Figure 3: Intraocular pressure (IOP) during the 5 year follow up
